# Ear biometrics for patient identification in global health: a field study to test the effectiveness of an image stabilization device in improving identification accuracy

**DOI:** 10.1186/s12911-019-0833-9

**Published:** 2019-06-18

**Authors:** Lauren P. Etter, Elizabeth J. Ragan, Rachael Campion, David Martinez, Christopher J. Gill

**Affiliations:** 1 College of Engineering, Boston University, Boston, MA USA; 2 Department of Medicine, Section of Infectious Diseases, Boston Medical Center, Boston, USA; 3 Department of Global Health, Boston University School of Public Health, 801 Massachusetts Avenue, Boston, MA 02118 USA

**Keywords:** Ear biometrics, Identification, Patient identification, Global health, Public health, Electronic medical record, Image stabilization, Pattern recognition algorithm

## Abstract

**Background:**

In many low and middle-income countries (LMICs), difficulties in patient identification are a major obstacle to the delivery of longitudinal care. In absence of unique identifiers, biometrics have emerged as an attractive solution to the identification problem. We developed an mHealth App for subject identification using pattern recognition around ear morphology (Project SEARCH (Scanning EARS for Child Health). Early field work with the SEARCH App revealed that image stabilization would be required for optimum performance.

**Methods:**

To improve image capture, we designed and tested a device (the ‘Donut’), which standardizes distance, angle, rotation and lighting. We then ran an experimental trial with 194 participants to measure the impact of the Donut on identification rates. Images of the participant’s left ear were taken both with and without use of the Donut, then processed by the SEARCH algorithm, measuring the top one and top ten most likely matches.

**Results:**

With the Donut, the top one identification rate and top ten identification rates were 99.5 and 99.5%, respectively, vs. 38.4 and 24.1%, respectively, without the Donut (*P* < 0.0001 for each comparison). In sensitivity analyses, crop technique during pre-processing of images had a powerful impact on identification rates, but this too was facilitated through the Donut.

**Conclusions:**

By standardizing lighting, angle and spatial location of the ear, the Donut achieved near perfect identification rates on a cohort of 194 participants, proving the feasibility and effectiveness of using the ear as a biometric identifier.

**Trial registration:**

This study did not include a medical intervention or assess a medical outcome, and therefore did not meet the definition of a human subjects research study as defined by FDAAA. We did not register our study under clinicaltrials.gov.

**Electronic supplementary material:**

The online version of this article (10.1186/s12911-019-0833-9) contains supplementary material, which is available to authorized users.

## Background

Any public health, clinical, or research program that requires collecting and acting upon longitudinal data critically depends on accurately and repeatedly identifying individuals over time and space.

Consider a newborn baby girl in Boston. There, her routine well-child care is delivered seamlessly because she is easily identified using multiple identifiers: home address, birth certificate, name and date of birth, insurance card, social security number, etc. Now, imagine that same infant girl in rural Zambia. As in many low and middle-income countries (LMICs), she has no formal mailing address; she is too young to have been named (in Zambia, naming is often deferred until 6 weeks of age); her family is too poor to participate in the national insurance system; and since she was delivered at home, a still-frequent occurrence in many countries, her birth was never formally registered, leaving her without a birth certificate or social security number.

Given this reality, a Zambian infant’s well-child care is coordinated using an ‘Under Five Card’, a 4 × 5 inch hard stock paper trifold which remains the mother’s responsibility to retain. When cards are lost or degraded to illegibility, a child’s vaccination history, growth curves, HIV screening data, and acute care history are irretrievably lost. In Zambia, as in many LMICs, the lack of robust patient identification renders even routine medical care fragmented, redundant, porous, and inefficient, and is a major barrier to the effective management of preventable and chronic diseases.

Recent years have seen an explosion of interest in developing electronic medical records (EMRs) adapted for LMICs to help facilitate longitudinal care and transfer the burden of record retention from the patient back to the clinic [1–6]. However, the enormous potential of EMRs can only be realized once the problem of subject identification has been solved. This is far from a reality. With the growing ubiquity of smartphones with powerful processing capacity, mobile health (mHealth) applications (Apps) may be particularly suited to solving the patient identification problem [7, 8]. 

In Project SEARCH (Scanning EARs for Child Health), our multidisciplinary team of public health, engineering, and computer science faculty and students at Boston University (BU) has focused on solving the identification problem through pattern recognition analysis of biometric data, using ear morphology as the identifier. Biometric data offer distinct advantages over external identifiers. By definition, biometric features are intrinsic and cannot be lost, left at home, sold, or traded. And the choice of ears is logical, offering clear advantages over other biometric identifiers: finger prints are stigmatized by association with law enforcement; faces lack anonymity; and iris/retinal scans require magnification and external lighting, rendering them futile for infants. By contrast, ears are impersonal, easily imaged with a smartphone’s camera with little to no discomfort, and are as unique as fingerprints [9–19]. 

Former work under the scope of Project SEARCH has involved three studies. In the analog proof of concept, a 3-dimensional object (the ear) was rendered into a set of stereotypical digital features. In a blinded matching exercise, an individual ear allowed for high re-identification rates (83%), proving the ear identifiable even with a relatively crude, analog approach [20]. In the digital proof of concept evaluation, Project SEARCH created the first version of the SEARCH mobile app. The App was built around a powerful pattern recognition algorithm (PRA) called ‘Scale Invariant Feature Transform’ (SIFT), [21] and allows one to register a new subject, capture an image of the ear, render it into a digital matrix, add this to a library of digital renditions, and then later interrogate that library of renditions to obtain a match. [22] The results from this experiment provided context that the SEARCH App was viable, but highlighted clear limitations. Most notably, the experiment used a set of images taken from an on-line database and did not evaluate the App’s performance in the face of random error introduced by the image capture process. In the third experiment, the ‘real-world’ evaluation, a birth cohort of 50 infants at a local pediatrics office were enrolled in a study to assess the process of image capture and how this affected the App’s performance. The analysis yielded critical results: random error around image capture had a profound negative impact on the accuracy of identification resulting in an inadequate recapture accuracy of just 27.3% (unpublished). All previous work clearly highlighted that in order for this simplified, computationally-efficient method to work, there was a need to minimize sources of random variation during image capture.

Results from these previous experiments set the stage for the current study, which was conceived and conducted by a team of three Boston University undergraduate engineering students in fulfilment of their senior project requirement under mentorship from professors of engineering and public health. The twin goals of this study were: (1) to design an image stabilization device that would improve the rate of re-identification by neutralizing variables of lighting, distance, and angle of image capture; and (2) to test how the use of this device improved individual identification rates using the SEARCH algorithm.

## Methods

### Technological background and system architecture

Literature suggests that most ear biometric identification processes make use of 2D feature extraction techniques [13, 22, 23]. Under previous work through Project SEARCH, a student team from the Computer Science Department at Boston University analyzed three common feature extraction methods and their accuracy when applied to ear biometric identification: Local Binary Patterns (LBPs), Generic Fourier Descriptor (GFD), and Scale Invariant Feature Transform (SIFT). To analyze these methods, the SEARCH team used 493 ear images of 125 subjects from the IIT Delhi Ear Image Database, and found that SIFT gave the highest accuracy in identifying the top 1 match correctly (96.5%) [22]. Since images were taken from an ear image database, where conditions of illumination and out of plane image capture angle are non-variable, this initial analysis did not take into account variables present in the field. Project SEARCH used the SIFT algorithm in the form of an iOS application, to test on a cohort of 50 infants in a clinical setting. In the face of random error, recapture rate plummeted to 27.3% (unpublished). The need to control for random error inherent in field settings set the stage for our current experiment.

**Fig. 1:**
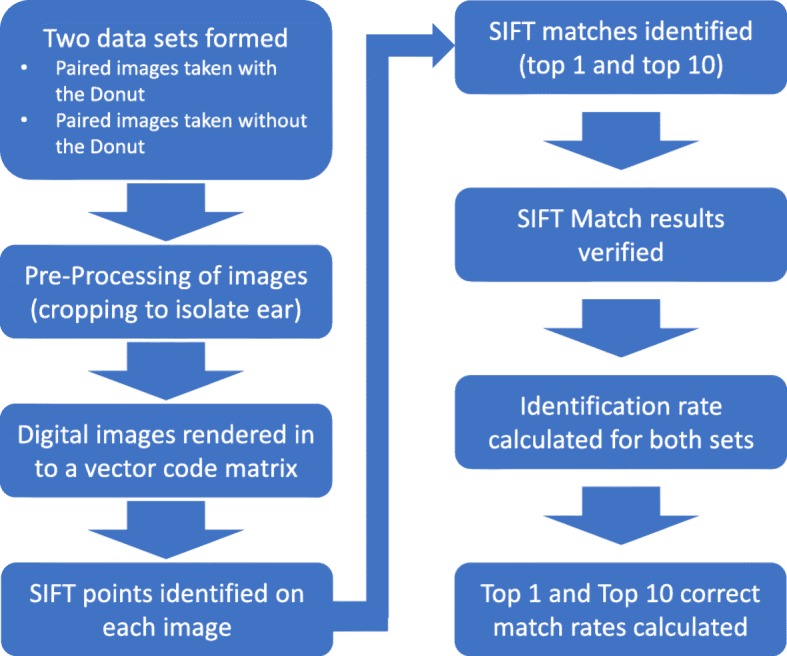
This figure depicts high-level system architecture of the Matlab algorithm used in our experiment

For our experiment, we use the Matlab algorithm developed in that initial study (with few modifications) in order to test the effect that neutralizing random error in the image capture process has on identification rates. The algorithm reads in two datasets of images (denoted first visit and second visit images, respectively), which are passed in as single precision, greyscale images. The images undergo a preprocessing step to resize and auto-crop all images passed into the system. Preprocessing limits the number of pixels that the open-source SIFT functions analyze, drastically cutting down processing time, which simulates implementation in a mobile application. After preprocessing, the algorithm reads in the first visit images to establish the cohort, and loops through the second visit images independently. A best (top one) match and a top ten list of matches for each image are determined based on a set of SIFT features, i.e. points of high contrast in the image. Each image is designated a SIFT feature vector, and the match is determined using a function that compares SIFT feature vectors. After all second visit images are read in and matched to images in the first visit dataset, the algorithm determines if the correct match was made. The correct match is determined by a function that checks if the matches made (both top one and top ten, independently) had the same image name. The name assigned to the images correspond to the participant number, and thus are the same in each dataset. An identification rate is computed (for both a top 1 match and a top 10 match), and the process repeats, using the second visit images to establish the cohort, looping independently through first visit images. The average of both processes is taken as the overall identification rate, yielding an averaged top match identification rate, and an average top ten identification rate. The algorithm runs twice, once yielding identification rates for images taken with the Donut (standardizing variables in the image capture process) and the second time yielding identification rates for images taken without the Donut (random error present). Fig. 1 provides a visual representation of the algorithm; access to the Matlab functions and main script are provided as Additional file 1.

### Image stabilization device design – creating the ‘Donut’

Although SIFT is considered a robust pattern recognition algorithm (controlling for angle and illumination variability fairly well when compared to other methods of pattern recognition), it was clear from SEARCH’s previous experiments that images generated in a lab setting and run through the SIFT algorithm outperformed images taken in the field, where angle and illumination varied greatly between images. Recreating lab conditions from field-generated images through image processing techniques is difficult to do when dealing with clinical field settings that are unpredictable in nature. For example, movement of the patient, ambient lighting changes, and hair occlusion are some of the many factors present in the field that are unpredictable and hard to control for using image processing techniques alone. Because of this, our approach was to create a simple image stabilization system that is used during the image capture process to create lab-like conditions. In order to create an effective stabilization system for the phone during image capture, we designed and manufactured a device - a lightweight opaque cylinder with a platform on the back to attach a smartphone, a hole to allow for the phone’s camera, and an internal 360-degree light source using energy-efficient light emitting diode (LED) strips. Understanding the use context of the device heavily informed the design process. A set of design requirements to minimize variables affecting the image capture process were developed from research, feedback from previous experiments, and heavy communication with members of the larger Project SEARCH team. Device requirements included: lightweight, accommodating to a range of ear sizes, able to standardize lighting of the image (by neutralizing external light and providing a constant and consistent source of internal light), sustainably powered, controlled for angle rotation, allowing of the attachment of varying sized phones, and aesthetically appropriate. After identifying the most important elements of the design, we created weighted metrics to rank each design feature. Informed decisions were guided by Pugh charts (Table 1), our chosen tool for alternate design analysis [24].

**Table 1:** Pugh Chart for Alternate Design Analysis

		Concepts		
Criteria	Weight	Diffused LED plate	Electroluminescent Ribbons	LED Strip
Shadow	2	0	0	+
Power Required	1	0	0	–
Shape	1	0	+	+
Buy/Make	1	0	0	+
Price	1	0	–	0
	Weighted Total	0	0	3

This Pugh chart was used to rank lighting designs for our Donut. Key metrics are weighted according to importance (2 being a more important design feature than 1). We used the diffused LED design as a baseline (all zero ranks). A positive mark indicates that the design outperforms the diffused LED for the specific metric, while a negative mark indicates underperformance. Weighted totals were determined by replacing the positive and negative signs with positive and negative one values, and multiplying each value by the corresponding metric’s weight. All values in each column were added, and totals for each design option were compared. The LED strip, with the highest total (3), was deemed the best lighting design option for the Donut

### Device manufacture method

**Fig. 2:**
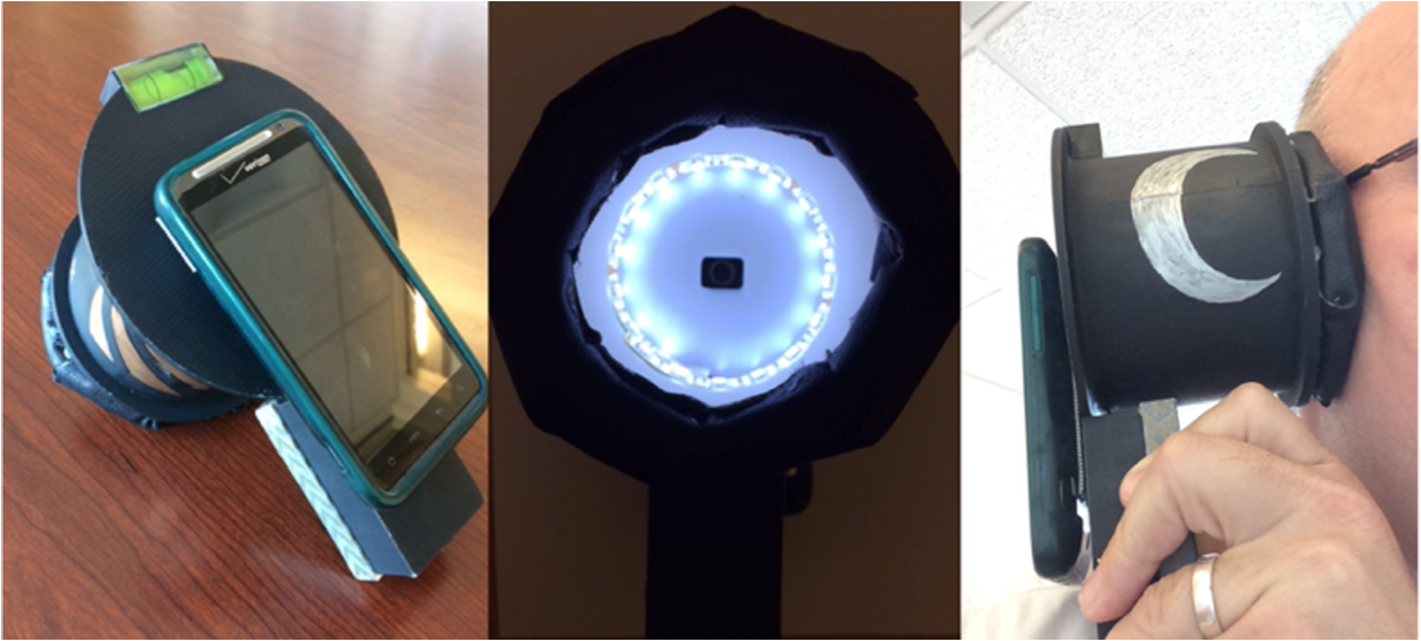
This figure shows the image stabilization device (the Donut). The leftmost image shows the back of the Donut, where the phone is attached. The bubble level is mounted on the top of the back of the Donut to control for angle rotation during image capture. The middle image shows looking into the Donut, the LED strip is laid along the inner base of the Donut. The right image demonstrates use of the Donut. The phone is mounted on the left, while the Donut interfaces with the participant on the right

The Donut was first modeled using a Computer Aided Design software, CREO. This model was converted to an STL file, adjusting chord length and angle for optimal 3D printing. The Donut was manufactured using a Uprint 3D printer, painted black to neutralize external light, and then assembled with the remaining design features. The circuit was secured to the bottom circumference of the Donut. Foam padding with an antimicrobial leather covering were attached to the top circumference (the area in contact with the patient’s head) in order to provide comfort and sterility. Finally, a bubble level (to standardize rotation of image capture) and Velcro (to easily mount the phone) were adhered to the back of the Donut. The final version that advanced to field testing is shown in Fig. 2.

### Clinical study methodology

To validate the impact of the Donut on identification accuracy, we conducted a field study among BU undergraduate students, all of whom provided informed consent. This involved creating a cohort and database of ear images taken both with and without use of the Donut. After receiving IRB approval, the cohort was established by contacting a number of different student organizations across Boston University’s campus.

To simulate two distinct patient visits at a clinic, we constructed our experimental study to capture two sets of pictures of each participant’s ear under different lighting conditions. To accomplish this, we stationed one study coordinator on one side of a classroom, and stationed a second study coordinator across the room facing a different direction (altering the ambient light). The participant visited the first study coordinator, where two images of their left ear were captured (one with the Donut and one without). These two images taken under identical ambient light, were designated ‘first visit’ images for the corresponding datasets (with and without Donut). The participant then walked to the second study coordinator where the process was repeated – designating ‘second visit’ images for the corresponding datasets (with and without Donut). A total of four images of each participant’s left ear were collected - two images taken with the Donut (first and second visit images), and two images taken without the Donut (first and second visit images).

### Power assumptions and statistical analysis

Based on Project SEARCH’s prior analog and digital proof of concept studies, we conservatively estimated the probability of a correct match at 80%: assuming that the Donut improved identification by 10%, we would need 199 participants. A type 1 error for the null hypothesis was 0.05 with 80% power. We used an uncorrected chi squared analysis to evaluate the null hypothesis that our device did not improve the identification accuracy. Once identification accuracies were determined, we used an uncorrected chi squared analysis to determine the statistical significance of using the Donut to aid the image capture process.

In order to analyze data from our study, we modified the previously developed Matlab algorithm used by SEARCH [22]. After running an image through the entire database, an ‘identification’ or match is determined, which corresponds to the image stored in the database that has the most matching SIFT points, i.e., points of high contrast [21, 23].

Two distinct identification accuracies, equivalent to match probability, were determined by running the Matlab algorithm twice – once using all images taken with the Donut and once using all images taken without the Donut. To analyze images taken with the Donut, two data sub-sets (‘first visit’, with Donut; ‘second visit’, with Donut) were passed into the Matlab algorithm as single-precision, greyscale images. A pre-processing step was done, using built in Matlab functions, to crop and resize all images. Pre-processing limited the number of pixels that the algorithm analyzed, which drastically cut down processing time, allowing the algorithm to simulate the speed needed to be effectively implemented in a mobile application. We tested a number of different dimensions in order to determine the ideal crop for images both with and without the device, respectively.

Next, the algorithm established the ‘first visit’ images as the database, and looped through the ‘second visit’ images independently, finding a best (top one) match and a top ten list of matches for each image, ranked in order of most likely match. After all ‘second visit’ images were read in and matched to images in the database, the algorithm determined: 1) if the correct match was made (top one), or 2) if the correct match resided within the top ten most likely matches (top ten).

An identification accuracy was computed (for both a top one match and a top ten match), and the process was repeated using the ‘second visit’ images as the database, and looping independently through the ‘first visit’ images. The average of both processes was taken as the overall identification accuracy, yielding an averaged top match identification accuracy, and an averaged top ten identification accuracy.

In order to most accurately compare improvement due to solely lighting and angle we analyzed the bias introduced by an automated cropping methodology. We completed a sensitivity analysis, with the goal of assessing whether or not pre-processing the images had a different effect between the two image datasets (with and without use of Donut). To accomplish this, we ran the algorithm multiple times, changing both the crop length (corresponding to ear length) and the crop width (corresponding to ear width) of images taken with and without the Donut. We analyzed how changing these variables affected the output of top one identification accuracy. Sensitivity was quantified as the change in identification accuracy with respect to change in crop. If sensitivity values were large (> 1), we deemed that the identification accuracy was largely affected by the crop. If values were largely different between the two datasets (with and without Donut), this would signify that pre-processing introduced bias between the datasets.

## Results

### Creating the Donut

Internal lighting of the device was deemed the most important design feature. Without a way to control for variable lighting conditions, we would be unable to improve the signal-to-noise ratio, thus the rate of re-identification. An LED strip inlaid along the inner circumference of the device proved the best solution for standardizing internal lighting, notably reducing shadow more than alternatives, such as a diffused LED plate or electroluminescent ribbons. To achieve optimal lighting, we powered the device with a 9 V battery – chosen due to its optimized voltage for the LED strip, availability, and it being an easily replaceable power supply. In order to maintain consistent illumination, we wired the battery in series with a 33-Ω resistor, which served as a voltage regulator. As the battery runs down, light intensity stays constant above a critical threshold and then switches off (rather than slowly dimming, as with the common household flashlight). Dimensions of the device were determined from research on the focus length of cell-phone cameras and the average size of the human ear. [25] These dimensions, which define the inner diameter and the depth of the device, were constrained by the average skull size of an infant (4 in.) [26]. The final specifications for the device are shown in Table 2. Aesthetic appropriateness of the Donut was achieved by employing similar methodology used in the GE Adventure Series (Fig. 2**)** [24], [27, 28].

**Table 2:** Final Device Dimensions

	Inner Diameter	Depth	Battery Box Width	Battery Box Height	Battery Box Length
Dimension (mm)	89	81	41	29	67.5

### Evaluation of the impact of the Donut on identification rates

Complete image sets were obtained from 194 participants enrolled in the study. Cohort demographics are summarized in Table 3**.** The majority of participants were Caucasian (62%) and male (60%). All were undergraduate students at Boston University who had been invited to participate after SEARCH team members made brief presentations at classes and to on campus student groups.

**Table 3:** Demographics of cohort

Variables	*n* = 194	
Race, %	Caucasian	120 (62%)
	Asian	45 (23%)
	Hispanic	13 (7%)
	African American	6 (3%)
	Other	10 (5%)
Sex, %	Male	116 (60%)
	Female	78 (40%)

This table identifies the demographics of our 194 participant cohort. Both race and gender demographics are broken down by number and percent

After processing the images through the MatLab algorithm, the following identification accuracies were determined. Images taken with use of the Donut yielded a top one match accuracy of 95.9% and a top 10 match accuracy of 99.5%, significantly outperforming the matching rates without the Donut (Table 4).

**Table 4:** Identification accuracies with and without use of the Donut

	Matched within top 10 most likely individuals (*N* = 194 paired images)	Matched to top ranked individual (*N* = 194 paired images)
With Donut	99.5%	95.9%
Without Donut	38.4%	24.1%
*P*-value	P < 0.0001	P < 0.0001

The identification accuracies are shown as percentages for each case: with Donut, top 10 accuracy; with Donut, top 1 accuracy; without Donut, top 10 accuracy; without Donut, top 1 accuracy

**Figs. 3:**
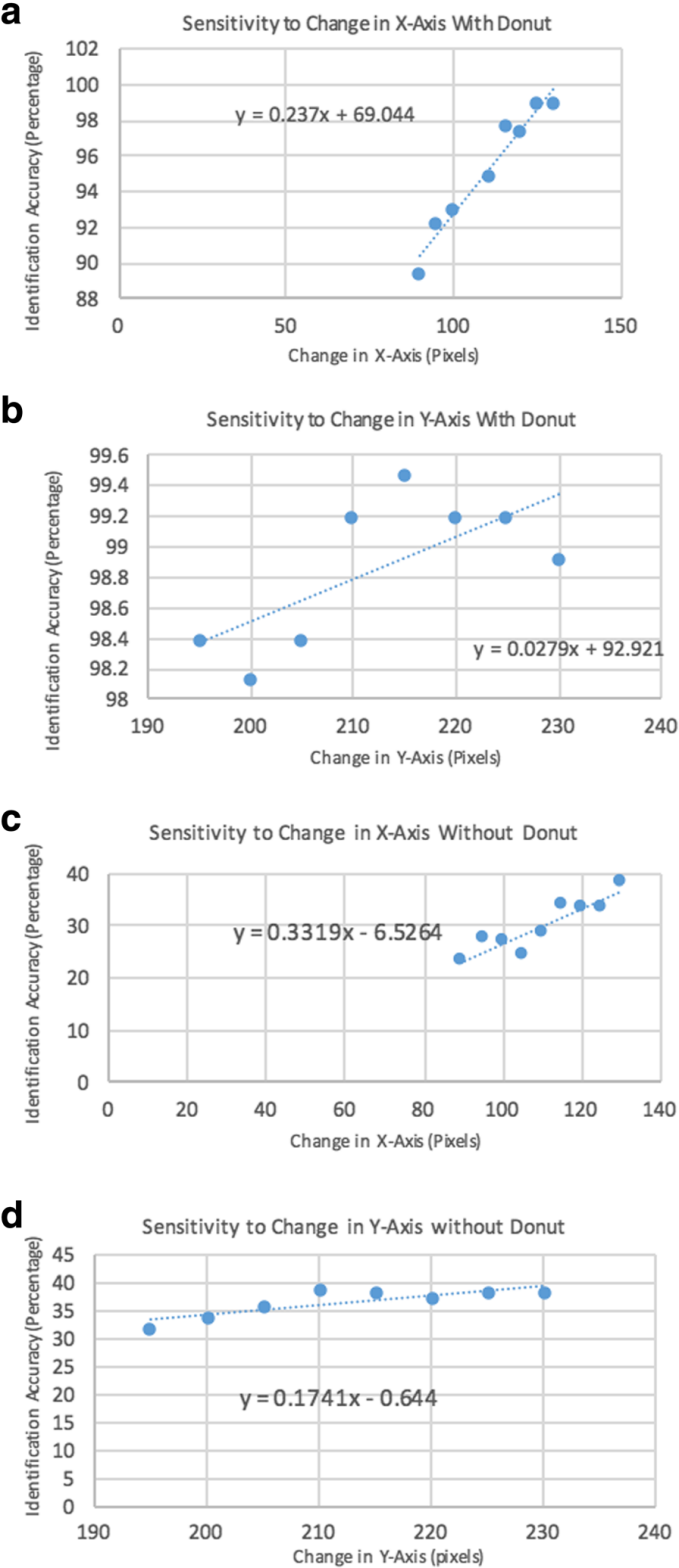
**a**-**d** These figures show the results of our sensitivity analysis. In each graph, x-axis corresponds to the dimension of the crop in pixels, while the y-axis corresponds to the identification accuracy. The data points were found by running the Matlab algorithm multiple times using a number of crop dimensions. The slope of each graph represents how the sensitivity of identification accuracy reacted to changing the crop dimension

A sensitivity analysis was performed to determine what effect pre-processing the images had on identification rates. Figures 3a-d outline the results of the sensitivity analysis. In both cases (with and without the Donut), identification accuracy was most sensitive when changing the length of the crop (corresponding to cropping the length of the ear), while cropping the width of the ear had little to no effect on identification accuracies. There was little difference in sensitivity values between the two datasets.

Additionally, we found that the Donut localized the ear during image capture. In order to be processed efficiently by the MatLab algorithm, images must be cropped and resized. For images taken with the Donut, we created an automatic crop in MatLab based on the edges of the Donut in the image. For images taken without the Donut, however, the ear was in a number of different spatial locations, making automatic crop dimensions difficult to determine and leading to cropping of parts of the image containing the ear, decreasing the identification accuracy.

## Discussion

In this experiment, we showed that the SEARCH algorithm’s performance was significantly improved with the use of an image stabilization/standardization device (i.e., the Donut). A direct comparison of the results from this experiment show that, with the device, we improved the identification accuracy from just under 40 to > 96% for the single most likely match, and to > 99% within the top 10 most likely matches. A previously completed study was able to get an accuracy of 80%, without any device, by employing a box on the screen of the phone in which a user would place the ear. Using this prior study as our benchmark expected value of 80%, our current results significantly exceeded our expectations. As hypothesized, quality control around image capture, and the ability to standardize lighting, distance, centering, rotation, and angle of capture, is a key determinant of the accuracy of biometric matching via pattern recognition algorithms and is a problem that is readily addressed using our simple mechanical engineering solution. We view this as a major step forward in Project SEARCH, and one that allows us to move forward with improving our design in future iterations.

Over the course of this experiment, we came across a number of notable findings. First, we found that hair occlusion within the image, in addition to lighting and rotation angle, was another factor that interfered with the image capture and identification process. While we had not planned this explicitly, it turned out that the design of the Donut tended to keep hair out of the image by consistently enclosing the ear during image capture. This is important because the pattern recognition algorithm is sensitive to all elements within the image, and hair, being mobile and therefore impossible to standardize, could be a source of a significant degree of random error. This unforeseen advantage to our device further limited the variables involved in the image capture process, likely contributing to the increased identification accuracy on images taken with our device. The implication is that future iterations of the Donut should include some method to systematically and more effectively exclude hair from the image, perhaps by including an internal shroud that would cup and therefore isolate the ear from the hair, head and neck, limiting the data for the SIFT algorithm to information just from the ear.

Additionally, through pre-processing the images, we found that our Donut localized spatial location of the ear, while images taken without the Donut did not. Because of this, automatic crop dimensions were determined for each dataset, independently. Since the Donut localized the ear during image capture, automatic crop dimensions were easier to determine in comparison to images taken without the Donut. This further emphasizes the need to standardize how the image is centered on the ear during capture, a problem that could be solved by software modifications to the SEARCH App (e.g., by including an on-screen C-shaped guided to align with the edge of the ear while taking the image), through post-capture automatic processing of the image, through design modifications to the Donut, or potentially all three.

Although our experiment was successful in many ways, there were clear limitations to our study that need to be addressed in future work. Major limitations to our methodology include that 1) this experiment was not done using an infant cohort, 2) identification accuracies were determined using a computer algorithm, not a fully developed smartphone application, and 3) there are newer computational methods that should also be considered and compared to SIFT’s performance. First, the most rapid period of ear growth occurs within the first few years of an infant’s life, and the algorithm has not been validated in a longitudinal field study on a cohort of infants. Validation in this context is a clear next step for testing the SEARCH platform. Second, our experiment tested identification rates using an algorithm after all data had been collected, and did not make use of a developed smartphone application. The application would need to be fully developed and tested in the field to prove that the capabilities of the application match that of the computer algorithm. Planning of a pragmatic field study in Zambia by assembling a longitudinal cohort of infants is currently underway. Lastly, these experiments used SIFT as the pattern recognition algorithm of choice. Since the start of SEARCH, there have been several advancements in the development of deep learning models in the field of computer vision. It should be noted that these models, although requiring a large amount of data to train, have the potential to improve performance in ear recognition. As research continues under Project SEARCH, an effort to explore these models is underway.

## Conclusions

Our experiment adds to previous work done through Project SEARCH aimed at proving ear biometrics an accurate and reliable method of patient identification. We demonstrated viability of the SEARCH App in a real-world context, and proved that image standardization and stabilization through a device could minimize random error during image capture and thereby improve accuracy. This project has proven that our device is both necessary and effective for identifying individuals using a SIFT biometric recognition algorithm. In the future, by combining our device with an application, Project SEARCH will have a cost effective, accurate way to identify and link patients to their medical records in Zambia.

## Additional file

Additional file 1: Matlab functions and main script. (ZIP 4 kb)

## Data Availability

The datasets used and/or analyzed during the current study are available from the corresponding author on reasonable request.

## Abbreviations

App: Application
BU: Boston University
CREO: A computer aided design software
LED: light emitting diode
LMIC: Low and Middle-Income Countries
mHealth: mobile health
SEARCH: Scanning Ears for Child Health
SIFT: Scale-Invariant Feature Transform

## References

[1] Mitchell M, Hedt-Gauthier BL, Msellemu D, Nkaka M, Lesh N. Using electronic technology to improve clinical care - results from a before-after cluster trial to evaluate assessment and classification of sick children according to Integrated Management of Childhood Illness (IMCI) protocol in Tanzania. BMC Med Inform Decis Mak. 2013;13:95. Epub 2013/08/29. 10.1186/1472-6947-13-95. PubMed PMID: 23981292; PubMed Central PMCID: PMCPMC3766002. 10.1186/1472-6947-13-95 PMC3766002 23981292

[2] Tapsfield JB, Jane Bates M. Hospital based palliative care in sub-Saharan Africa; a six month review from Malawi. BMC Palliat Care. 2011;10:12. Epub 2011/07/12. 10.1186/1472-684X-10-12. PubMed PMID: 21740584; PubMed Central PMCID: PMCPMC3146929. 10.1186/1472-684X-10-12 PMC3146929 21740584

[3] Oluoch Tom, Santas Xenophon, Kwaro Daniel, Were Martin, Biondich Paul, Bailey Christopher, Abu-Hanna Ameen, de Keizer Nicolette (2012). The effect of electronic medical record-based clinical decision support on HIV care in resource-constrained settings: A systematic review. International Journal of Medical Informatics.

[4] Ali Mohammed K., Shah Seema, Tandon Nikhil (2011). Review of Electronic Decision-Support Tools for Diabetes Care: A Viable Option for Low- and Middle-Income Countries?. Journal of Diabetes Science and Technology.

[5] Hotchkiss DR, Diana ML, Foreit KG. How can routine health information systems improve health systems functioning in low- and middle-income countries? Assessing the evidence base. Adv Health Care Manag 2012;12:25–58. Epub 2012/08/17. PubMed PMID: 22894044. 10.1108/s1474-8231(2012)0000012006 22894044

[6] Piette John, Lun KC, Moura Lincoln, Fraser Hamish, Mechael Patricia, Powell John, Khoja Shariq (2012). Impacts of e-health on the outcomes of care in low- and middle-income countries: where do we go from here?. Bulletin of the World Health Organization.

[7] van Velthoven MH, Car J, Zhang Y, Marusic A. mHealth series: new ideas for mHealth data collection implementation in low- and middle-income countries. J Glob Health 2013;3(2):020101. Epub 2013/12/24. 10.7189/jogh.03.020101. PubMed PMID: 24363911; PubMed Central PMCID: PMCPMC3868820. 10.7189/jogh.03.020101 PMC3868820 24363911

[8] van Heerden Alastair, Tomlinson Mark, Swartz Leslie (2012). Point of care in your pocket: a research agenda for the field of m-health. Bulletin of the World Health Organization.

[9] Jain A.K., Ross A., Prabhakar S. (2004). An Introduction to Biometric Recognition. IEEE Transactions on Circuits and Systems for Video Technology.

[10] Burge M, B W. Ear biometrics in computer vision. Proceedings of the international conference on pattern recognition. 2000:822–6.

[11] Iannerelli AV (1989). Ear identification (forensic identification series). Paramount publishing company.

[12] Yan P, KB. Ear biometrics using 2D and 3D images. Proceedings IEEE Conference Computer Vision and Pattern Recognition Workshop in Advanced 3D imaging for safety and security2005. p. 121.

[13] Chang K, Bowyer K, Sarkar S, B. V. Comparison and combination of ear and face images in appearance-based biometrics. IEEE Transactions Pattern Analysis Machine Intelligence2003. p. 1160–65.

[14] Moreno B, AS. On the use of outer ear images for personal identification in security applications. Proceedings of IEEE 33rd annual international conference on Secruity Technology1999. p. 469–76.

[15] Pun K, YM. Recent advances in ear biometrics. Proceedings of the sixth IEEE International Conference on automatic face and gesture recognition2004. p. 164–69.

[16] Khursheed F, AM. time series model based personal verification using ear biometrics. 4th international conference on computer and communication Technology2013. p. 264–68.

[17] Ibrahim MI, Nixon MS, SM. The effect of time on ear biometrics. IEEE 2011;2011.

[18] Pflug A., Busch C. (2012). Ear biometrics: a survey of detection, feature extraction and recognition methods. IET Biometrics.

[19] Abaza A, Ross A, Heber C, Harrison MAF, MSN. A survey of ear biometrics. In: Surv AC, editor.2013. p. 1–35.

[20] Ragan EJ, Johnson C, Milton JN, Gill CJ. Ear biometrics for patient identification in global health: a cross-sectional study to test the feasibility of a simplified algorithm. BMC Res Notes 2016;9(1):484. Epub 2016/11/04. 10.1186/s13104-016-2287-9. PubMed PMID: 27806727; PubMed Central PMCID: PMCPMC5094067. 10.1186/s13104-016-2287-9 PMC5094067 27806727

[21] Introduction to SIFT (scale-invariant feature transform) [April 2017]. Available from: http://opencv-python-tutroals.readthedocs.io/en/latest/py_tutorials/py_feature2d/py_sift_intro/py_sift_intro.html.

[22] Bargal S.A. WA, Chan C. R., Howes S., Sclaroff S., Ragan E., Johnson C., Gill C.J. Image-based ear biometric smarphone app for patient identification in field settings. International Conference on Computer Visiion Theory and Application (VISAPP); Berlin, Germany2015.

[23] Li Dong, Zhou Huiling, Lam Kin-Man (2015). High-Resolution Face Verification Using Pore-Scale Facial Features. IEEE Transactions on Image Processing.

[24] Burge S. The systems engineering toolbox 2018 [May 2018]. Available from: https://www.burgehugheswalsh.co.uk/uploaded/1/documents/pugh-matrix-v1.1.pdf.

[25] Niemitz Carsten, Nibbrig Maike, Zacher Vanessa (2007). Human ears grow throughout the entire lifetime according to complicated and sexually dimorphic patterns - conclusions from a cross-sectional analysis. Anthropologischer Anzeiger.

[26] Nellhaus G. Head circumference from birth to eighteen years. Practical composite international and interracial graphs. Pediatrics. 1968;41(1):106–14. Epub 1968/01/01. PubMed PMID: 5635472. 5635472

[27] Brucker Michael J., Patel Jagruti, Sullivan Patrick K. (2003). A Morphometric Study of the External Ear: Age- and Sex-Related Differences. Plastic and Reconstructive Surgery.

[28] Adventure series for CT [April 2018]. Available from: https://www.gehealthcare.com/products/accessories-and-supplies/adventure-series-for-ct.

